# A nationwide causal mediation analysis of survival following ST-elevation myocardial infarction

**DOI:** 10.1136/heartjnl-2019-315760

**Published:** 2019-11-15

**Authors:** Tatendashe Bernadette Dondo, Marlous Hall, Theresa Munyombwe, Chris Wilkinson, Mohammad E Yadegarfar, Adam Timmis, Philip D Batin, Tomas Jernberg, Keith AA Fox, Chris P Gale

**Affiliations:** 1 Clinical and Population Sciences Department, Leeds Institute of Cardiovascular and Metabolic Medicine, University of Leeds, Leeds, UK; 2 NIHR Cardiovascular Biomedical Research Unit, Barts Health NHS Trust, London, UK; 3 Department of Cardiology, Pinderfields General Hospital, Wakefield, UK; 4 Department of Medicine, Section of Cardiology, Karolinska University Hospital, Huddinge, Karolinska Institutet, Stockholm, Sweden; 5 Department of Cardiology, University of Edinburgh and Royal Infirmary of Edinburgh, Edinburgh, UK

**Keywords:** acute myocardial infarction, epidemiology, electronic medical records, quality and outcomes of care, acute coronary syndromes

## Abstract

**Objective:**

International studies report a decline in mortality following ST-elevation myocardial infarction (STEMI). The extent to which the observed improvements in STEMI survival are explained by temporal changes in patient characteristics and utilisation of treatments is unknown.

**Methods:**

Cohort study using national registry data from the Myocardial Ischaemia National Audit Project between first January 2004 and 30th June 2013. 232 353 survivors of hospitalisation with STEMI as recorded in 247 hospitals in England and Wales. Flexible parametric survival modelling and causal mediation analysis were used to estimate the relative contribution of temporal changes in treatments and patient characteristics on improved STEMI survival.

**Results:**

Over the study period, unadjusted survival at 6 months and 1 year improved by 0.9% and 1.0% on average per year (HR: 0.991, 95% CI: 0.988 to 0.994 and HR: 0.990, 95% CI: 0.987 to 0.993, respectively). The uptake of primary percutaneous coronary intervention (PCI) (HR: 1.025, 95% CI: 1.021 to 1.028) and increased prescription of P2Y_12_ inhibitors (HR: 1.035, 95% CI: 1.031 to 1.039) were significantly associated with improvements in 1-year survival. Primary PCI explained 16.8% (95% CI: 10.8% to 31.6%) and 13.2% (9.2% to 21.9%) of the temporal survival improvements at 6 months and 1 year, respectively, whereas P2Y_12_ inhibitor prescription explained 5.3% (3.6% to 8.8%) of the temporal improvements at 6 months but not at 1 year.

**Conclusions:**

For STEMI in England and Wales, improvements in survival between 2004 and 2013 were significantly explained by the uptake of primary PCI and increased use of P2Y_12_ inhibitors at 6 months and primary PCI only at 1 year.

**Trial registration number:**

NCT03749694

## Introduction

There has been a global decline in mortality and non-fatal complications following acute myocardial infarction.^1^ For ST-elevation myocardial infarction (STEMI), the adoption of new health technologies such as primary percutaneous coronary intervention (PPCI) as well as the availability of novel pharmacotherapies has been identified as driving improvements in clinical outcomes.^2^ However, the extent to which population-based temporal improvements in outcomes from STEMI are due to the uptake of, say, PPCI compared with other guideline-indicated treatments or changes in patient characteristics is not known. Resolving the knowledge gap around the effectiveness of STEMI treatments on temporal outcomes could help future healthcare planning for developing countries with, or predicted to have, a high burden of cardiovascular disease.

Notably, there is a paucity of large-scale cohorts that are of sufficient duration to enable a detailed evaluation of the association of baseline risk and guideline-indicated therapies with temporal trends in STEMI mortality.^3–5^ Where there have been studies of treatments and outcomes for STEMI, analyses have quantified associations and not necessarily reported explanatory (causal) factors. The Myocardial Ischaemia National Audit Project (MINAP) is a whole country registry of hospitalised cases of acute coronary syndrome (ACS), representing all hospitals in a single health system (the National Health Service of England and Wales) with prospective collection of detailed information about quality of care and clinical outcomes of patients for more than 15 years.^6 7^ Our objective was to investigate whether temporal improvements in survival were associated with changes in patients’ baseline clinical risk or use of guideline-indicated treatments for the management of STEMI, and to determine the extent to which associations explained the temporal improvements in survival.

## Methods

### Data and patients

The analyses were based on data from MINAP, a comprehensive registry of ACS hospitalisations started in 2000 and mandated by the Department of Health in England and Wales.^6 7^ Data were collected prospectively at each hospital, electronically encrypted and transferred online to a central database. Data entry is subject to routine error checking and a mandatory annual data validation exercise. Patient-level data concerning demographics, cardiovascular risk factors, medical history and clinical characteristics at the time of hospitalisation were extracted from MINAP and (if applicable) date of death from linkage to the Office for National Statistics. Further details of MINAP have been published elsewhere.^6 7^ The diagnosis of STEMI was based on guidelines from the European Society of Cardiology (ESC), American College of Cardiology and American Heart Association, and determined at local level by the attending Consultant on discharge from hospital.^8^

**Figure 1:**
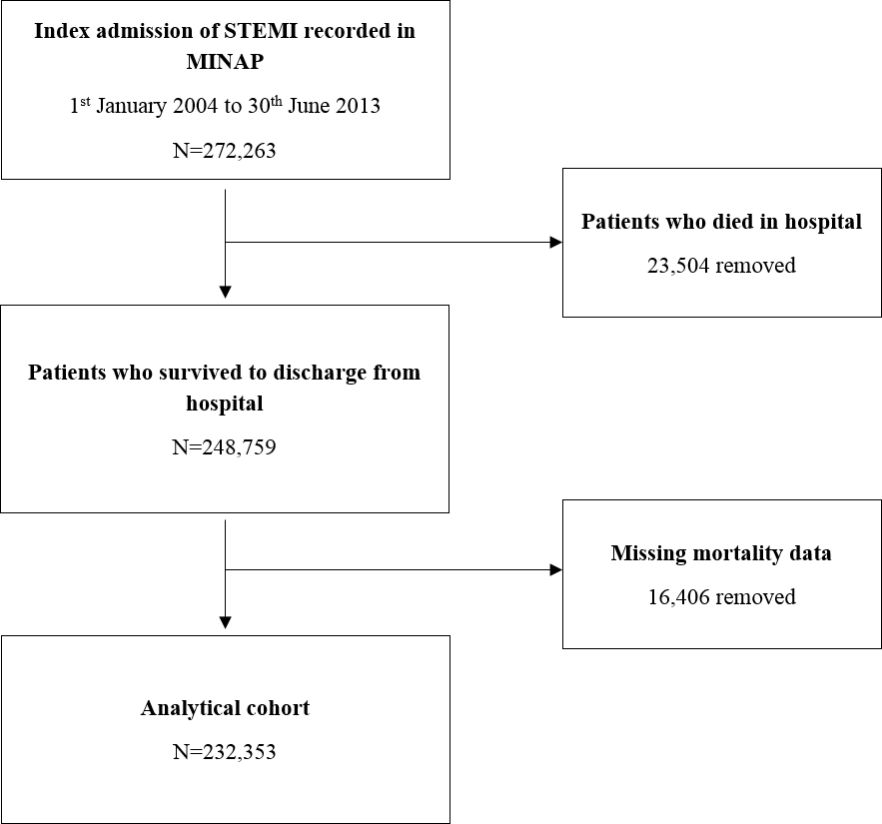
Strengthening the Reporting of Observational Studies in Epidemiology (STROBE) diagram showing the derivation of the analytical cohort from the Myocardial Ischaemia National Audit Project dataset.

The analytical cohort (n=232 353) was drawn from 272 263 patients with STEMI admitted to one of 247 hospitals between first January 2004 and 30 June 2013 (figure 1). For multiple admissions, we used the earliest record. As discharge medication was a key exposure, we excluded 23 504 (8.6%) who died in hospital; 16 406 (6.0%) patients with missing mortality data were also excluded. The primary outcome was all-cause mortality at 1 year following discharge from hospital. For care interventions, patients were classified as ineligible if a treatment was contraindicated, not indicated, not applicable, if the patient declined treatment as recorded in MINAP or if the admission preceded the inclusion of the treatment in guidelines (online supplementary eTable 1).

10.1136/heartjnl-2019-315760.supp1 Supplementary data

### Statistical analyses

Baseline characteristics were described using numbers and percentages for categorical data and means and SD or medians and interquartile ranges for normally and non-normally distributed continuous variables. Temporal trends of patient and treatment characteristics were summarised by comparing data from the start of the study (2004–2005) to the end of the study (2012–2013) using χ^2^ tests, t-tests and Wilcoxon rank sum tests.

Royston-Parmar flexible parametric survival models^9^ were fitted to explore the association between temporal changes in patient demographics (age, sex, socioeconomic deprivation (Index of Multiple Deprivation score), comorbidities and risk factors (diabetes, hypercholesterolaemia, hypertension, smoking status, asthma/chronic obstructive pulmonary disease, chronic renal failure, chronic cardiac failure, cerebrovascular disease, peripheral vascular disease, angina, previous myocardial infarction, PCI and coronary artery bypass graft surgery), pharmacological treatments (secondary prevention pharmacotherapies prescribed at hospital discharge: aspirin, statins, P2Y_12_ inhibitors, ACE inhibitors (ACEi)/ARBs and β-blockers), cardiac rehabilitation and reperfusion strategy (defined as receipt of PPCI) with temporal changes in 1-year survival from hospital discharge. Flexible parametric survival models were selected in favour of Cox regression models to overcome violation of the proportional hazards assumption. Improvements in model fit at each stage were determined by minimising the Akaike information criterion (AIC) and Bayesian information criterion (BIC) (online supplementary eTable 2). The scale (proportional hazards, proportional odds or normal) and complexity (number of degrees of freedom) for flexible parametric survival models were checked on the full multivariable model for each imputation. The baseline hazard on the normal scale with five degrees of freedom produced the optimal model through minimisation of the AIC and BIC (online supplementary eTable 2).

Initially, to determine the overall temporal trend in 1-year survival, an unadjusted model comprising only the year of admission was fitted. Subsequently, to determine the impact of patient demographics, PPCI, comorbidities and risk factors, aspirin at discharge, statin at discharge, P2Y_12_ inhibitors at discharge, ACEi/ARBs at discharge, β-blockers at discharge and cardiac rehabilitation on the temporal trend in survival, each of these factors were added individually to the univariate year model. These models were then adjusted for age, sex and deprivation to form the primary analysis.

Secondary analyses included; a full model including all of the explanatory and adjustment variables stated above. This strategy allowed the influence of each factor on the temporal survival trend to be assessed in both an unadjusted and adjusted manner. To confirm that the identified factors had an association with survival trends, two flexible parametric survival models were fit, one including only the subset of variables found to have an influence on the survival trends (model A) and the other including only the subset variables found not to have an influence on survival trends (model B).

A causal mediation analysis was conducted to determine the relative contribution of patient and treatment variables on the survival trend. This analysis allowed the assessment of potential causal pathways that linked year of admission to temporal improvements in STEMI survival, such as through changes in clinical factors or therapeutic strategies, beyond simple point estimates. The magnitude of the contribution of the mediating variables was adjusted for confounding variables (online supplementary section 2) and presented as proportions.

To mitigate potential bias caused by missing data, we used multiple imputation by chained equations to create 10 datasets from 20 iterations, of which the resultant model estimates for each were combined using Rubin’s rules (Section 3, eTable three in the Supplement for the imputation strategy). As a secondary outcome, we repeated the above analytical methods for temporal changes in 6-month survival. All tests were two-sided, and statistical significance was considered p<0.05. Statistical analyses were performed in Stata V.14 (http://www.stata.com/) and R V.1.2 (https://cran.r-project.org/bin/windows/base/).

### Patient involvement

This study did not involve patients/service users/carers/lay people in its design or for the development of outcome measures.

## Results

Of the analytical cohort (n=2 32 353), 72.0% (n=1 66 690) were men, and the median age was 64.6 (IQR 55.0–75.0) years. A high proportion had hypertension (37.9%, n=87 990), a family history of coronary heart disease (25.7%, n=59 709) or were current or ex-smokers (68.1%, n=1 43 508) (table 1). There were 12 143 (5.2%) deaths at 6 months and 16 239 (7.0%) deaths at 1 year after hospital discharge.

**Table 1:** Patient characteristics, by year of hospitalisation

Variable		2004–2013 n=2 32 353 (Total cohort)	2004–2005 n=42,799 (18.4% of the cohort)	2012–2013 n=37,081 (16.0% of cohort)	Difference between 2004–2005 and 2012–2013 (95% CI)	P value
Age (years)	Median (IQR)	64.6 (55.0–75.0)	65.3 (55.8–74.9)	64.0 (54.5–74.9)	1.30 (1.04 to 1.56)	<0.001
Sex (male)	N (%)	166 690 (72.0)	30 332 (71.2)	26 590 (72.2)	0.93 (0.30 to 1.56)	0.004
Deprivation (IMD score)	Median (IQR)	18.4 (10.5–32.2)	18.7 (10.6–32.6)	18.4 (10.5–32.1)	0.37 (0.08 to 0.66)	0.012
Systolic blood pressure (mm Hg)	Mean (SD)	136.5 (28.2)	139.9 (29.2)	132.8 (26.9)	−7.18 (−7.61 to −6.74)	<0.001
Heart rate (beats per min)	Mean (SD)	77.9 (20.8)	77.1 (21.4)	77.9 (19.4)	0.83 (0.51 to 1.14)	<0.001
Total cholesterol (mg/dL)	Median (IQR)	5.1 (4.2–6.0)	5.4 (4.5–6.3)	4.9 (4.0–5.8)	0.50 (0.47 to 0.53)	<0.001
Creatinine (mg/dL)	Median (IQR)	87.0 (74.0–104.0)	101.0 (90.0–116.0)	82.0 (70.0–99.0)	19.0 (17.3 to 20.7)	<0.001
Ejection fraction <50%	N (%)	38 634 (49.3)	1753 (53.2)	9598 (49.5)	−3.72 (−5.56 to −1.88)	<0.001
Medical history						
Previous diabetes	N (%)	29 083 (12.5)	5265 (12.3)	5158 (13.9)	1.61 (1.14 to 2.08)	<0.001
Current or ex-smoker	N (%)	143 508 (68.1)	27 098 (69.9)	22 338 (66.0)	3.93 (−4.61 to 3.25)	<0.001
Family history of CHD	N (%)	59 709 (25.7)	4633 (10.8)	10 249 (27.6)	16.8 1 (16.27 to 17.34)	<0.001
Hypertension	N (%)	87 990 (37.9)	15 938 (37.2)	13 960 (37.7)	0.41 (−0.26 to 1.08)	0.235
Previous MI	N (%)	26 892 (11.6)	5525 (12.9)	4012 (10.8)	−2.09 (−2.54 to −1.64)	<0.001
Previous angina	N (%)	31 060 (13.4)	7050 (16.5)	4036 (10.9)	−5.59 (−6.06 to −5.11)	<0.001
Peripheral vascular disease	N (%)	5868 (2.5)	1168 (2.7)	950 (2.7)	−0.17 (−0.39 to 0.06)	0.143
Cerebrovascular disease	N (%)	10 415 (4.5)	1896 (4.4)	1612 (4.4)	−0.08 (−0.37 to 0.20)	0.569
COPD or asthma	N (%)	23 404 (10.1)	4444 (10.4)	3745 (10.1)	−0.28 (−0.71 to 0.14)	0.187
Chronic renal failure	N (%)	4410 (1.9)	576 (1.4)	793 (2.1)	0.79 (0.61 to 0.98)	<0.001
Congestive cardiac failure	N (%)	3593 (1.6)	762 (1.8)	529 (1.4)	−0.35 (−0.53 to −0.18)	<0.001
Previous PCI	N (%)	12 006 (5.2)	1488 (3.5)	2318 (6.3)	2.77 (2.47 to 3.08)	<0.001
Previous CABG	N (%)	5217 (2.3)	921 (2.2)	840 (2.3)	0.11 (−0.09 to 0.32)	0.276
Admission diagnosis						
ACS or probable MI	N (%)	217 563 (93.6)	40 231 (94.0)	35 270 (95.1)	1.12 (0.80 to 1.43)	<0.001
Chest pain unknown cause	N (%)	6810 (2.9)	1313 (3.1)	797 (2.2)	−0.92 (−1.14 to 0.70)	<0.001
Other	N (%)	7976 (3.4)	1255 (2.9)	1014 (2.7)	−0.20 (−0.43 to 0.03)	0.093
Preadmission medications*						
Aspirin	N (%)	146 742 (64.4)	26 121 (62.9)	25 229 (68.8)	5.88 (5.22 to 6.55)	<0.001
β-blocker	N (%)	37 199 (22.3)	4294 (32.2)	6097 (20.3)	−11.94 (−12.85 to −11.0)	<0.001
Statin	N (%)	54 151 (31.2)	5008 (37.7)	9654 (30.2)	−7.47 (−8.44 to −6.50)	<0.001
ACEi or ARBs	N (%)	45 897 (27.5)	4264 (32.2)	8424 (28.0)	−4.20 (−5.14 to −3.25)	<0.001
P2Y_12_ inhibitor	N (%)	13 136 (14.2)	–	4566 (14.8)	–	
Warfarin	N (%)	6891 (3.7)	1558 (4.6)	1060 (3.6)	−1.00 (−1.31 to −0.69)	<0.001
Discharge medications*						
Aspirin	N (%)	186 098 (98.8)	35 753 (98.1)	30 197 (99.2)	1.13 (0.96 to 1.30)	<0.001
β-blocker	N (%)	165 472 (95.7)	30 375 (94.1)	28 207 (97.5)	3.40 (3.09 to 3.72)	<0.001
Statin	N (%)	185 710 (98.1)	35 708 (97.3)	30 029 (98.8)	1.46 (1.26 to 1.67)	<0.001
ACEi or ARB	N (%)	173 303 (95.3)	32 616 (93.1)	28 567 (97.3)	4.16 (3.83 to 4.48)	<0.001
P2Y_12_ inhibitor	N (%)	89 676 (96.7)	190 (94.5)	28 694 (97.6)	3.05 (−0.10 to 6.20)	0.005
Aldosterone antagonist	N (%)	7296 (12.6)	0 (0)	3031 (15.8)	–	–
Reperfusion strategy*						
PPCI	N (%)	83 627 (39.6)	148 (2.1)	26 799 (80.2)	78.15 (77.61 to 78.69)	<0.001
Thrombolysis	N (%)	83 800 (39.6)	30 220 (81.1)	1218 (15.6)	−65.54 (−66.44 to −64.65)	<0.001
CABG	N (%)	1638 (0.8)	276 (3.8)	247 (3.6)	0.17 (−0.79 to 0.45)	0.593
None†	N (%)	42 354 (20.0)	6955 (18.6)	6275 (18.2)	−0.40 (−1.00 to 0.13)	0.129
Cardiac rehabilitation	N (%)	182 575 (92.4)	33 933 (89.8)	30 387 (94.3)	4.44 (4.05 to 4.84)	<0.001
GRACE risk score category						
Lowest (<70)	N (%)	5034 (5.0)	30 (5.6)	1099 (4.7)	−0.84 (−2.79 to 1.11)	0.362
Low (70-87)	N (%)	11 541 (11.4)	61 (11.3)	2654 (11.4)	0.08 (−2.61 to 2.78)	0.952
Intermediate to high (≥88)	N (%)	84 509 (83.6)	450 (83.2)	19 612 (83.9)	0.76 (−2.43 to 3.95)	0.635
Crude mortality						
30 days	N (%)	5517 (2.4)	1046 (2.4)	836 (2.3)	−0.18 (−0.40 to 0.02)	0.078
6 months	N (%)	12 143 (5.2)	2347 (5.5)	1703 (4.6)	−0.89 (−1.19 to −0.59)	<0.001
1 year	N (%)	16 239 (7.0)	3221 (7.5)	2090 (5.6)	−1.89 (−2.23 to −1.55)	<0.001

*Only patients eligible (eligibility criteria definition used given in online supplementary section 1) to receive treatments were included in the denominator of the complete cases.

†Reasons for non-receipt of PPCI/thrombolysis included: (1) ineligible ECG (25.2% (n=10,678)), (2) too late (32.8% (n=13,878)), (3) risk of haemorrhage (6.7% (n=2,837)), (4) uncontrolled hypertension (0.5% (n=209)), (5) administrative failure (1.7% (n=711)), (6) elective decision (15.4% (n=6,521)), (7) patient refused treatment (1.4% (n=570)) and (8) other (16.4% (n=6,950)).

ACEi, ACE inhibitor; ACS, acute coronary syndrome; ARBs, angiotensin receptor blocker; CABG, coronary artery bypass grafting; CHD, coronary heart disease; COPD, chronic obstructive pulmonary disease; GRACE, Global Registry of Acute Coronary Events; IMD, index of multiple deprivation; MI, myocardial infarction; PCI, percutaneous coronary intervention; PPCI, primary percutaneous coronary intervention.

### Temporal trends in clinical characteristics

**Figure 2:**
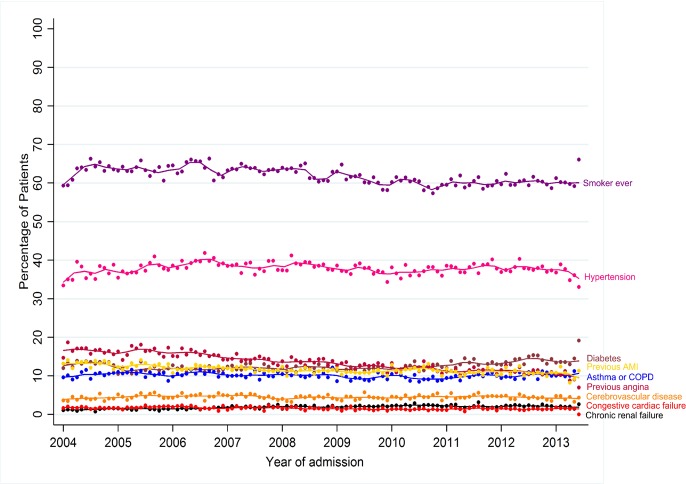
Temporal trends of comorbidities and risk factors, per month, 2004–2013.

Over the study period, the proportion of STEMI who had a previous myocardial infarction (12.9 vs 10.8%), angina (16.5 vs 10.9%) and were current or ex-smokers (69.9 vs 66.0%) decreased (all p<0.001), while the proportion of patients with diabetes (12.3 vs 13.9%), chronic renal failure (1.4 vs 2.1%) and previous PCI (3.5 vs 6.3%) increased (all p<0.001) (table 1 and figure 2). The proportion with a reduced ejection fraction (EF <50%) decreased from 53.2% in 2004-05 to 49.5% in 2012–2013 (table 1).

### Temporal trends in guideline-indicated treatments

**Figure 3:**
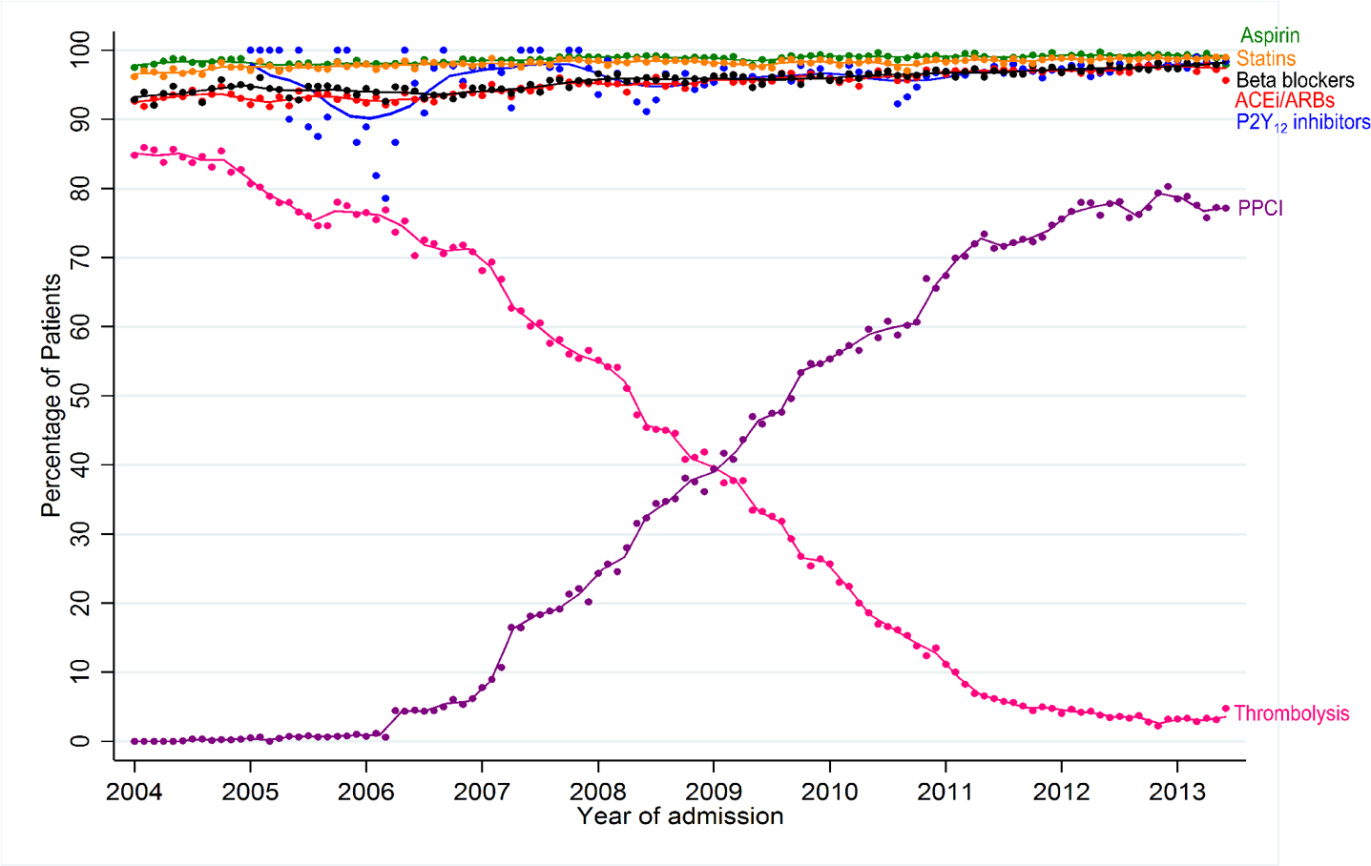
Use of pharmacological therapies at hospital discharge, thrombolysis and primary percutaneous coronary intervention per month, 2004–2013.

The use of the secondary prevention pharmacotherapies at hospital discharge was high (>90% for all five drugs), and increased over the study period: aspirin increased from 98.1% to 99.2% (difference 1.1%, 95% CI: 1.0% to 1.3%), β-blockers from 94.1% to 97.5% (difference 3.4%, 3.1% to 3.7%), statins from 97.3% to 98.8% (difference 1.5%, 1.3% to 1.7%) and ACEi/ARBs from 93.1% to 97.3% (difference 4.2%, 3.8% to 4.5%) (table 1 and figure 3). Overall, 39.6% (n=83 627) received PPCI and 39.6% (n=83 800) received thrombolysis. The majority (n=30 220, 81.1%) of those hospitalised in 2004–2005 received thrombolysis, and the majority of those hospitalised in the years 2012–13 received PPCI (n=26 799, 80.2%).

### Association between changing risk profile and improved outcomes

#### One-year survival

**Figure 4:**
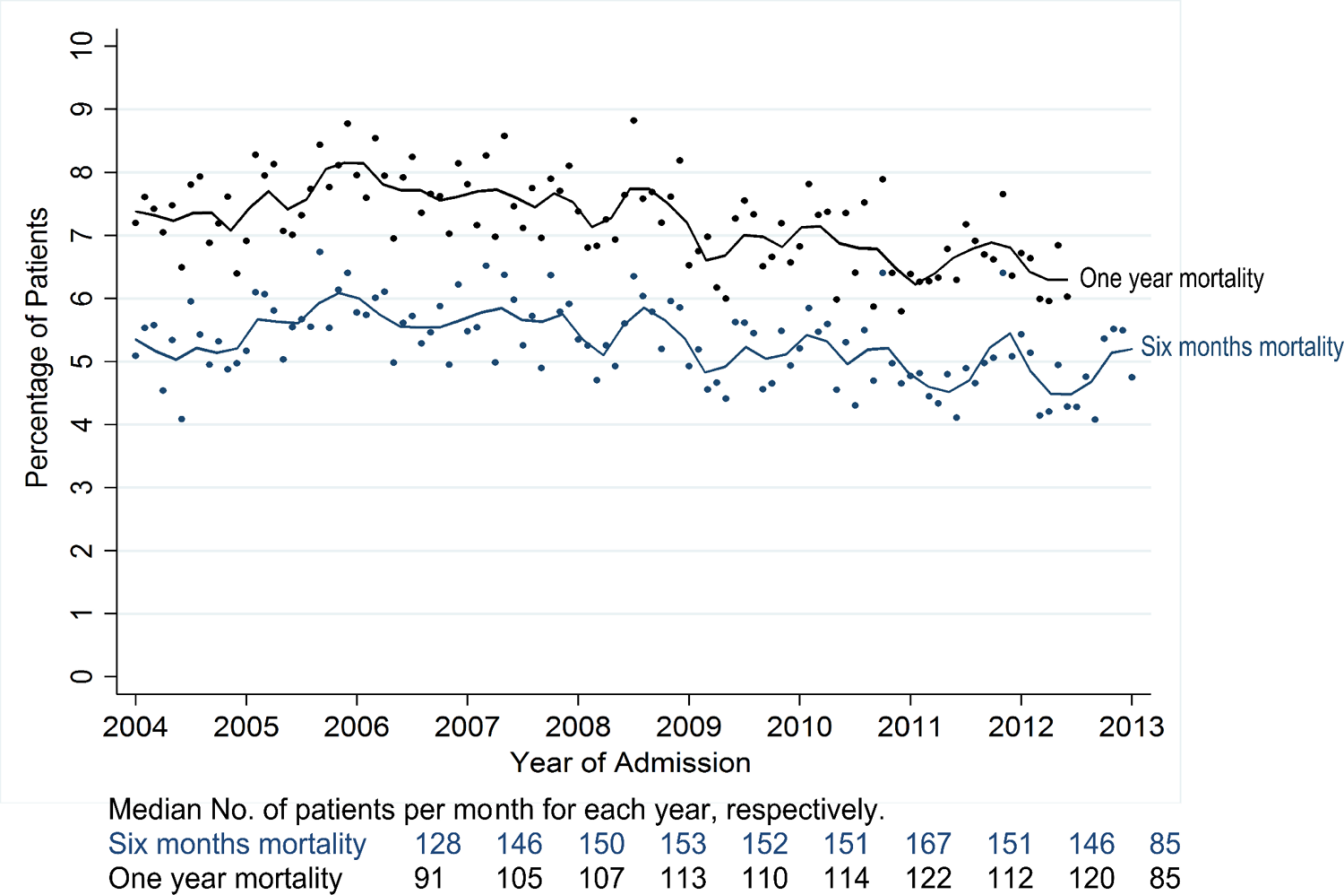
Crude all-cause mortality at 6-month and 1-year following hospital discharge, per month, 2004–2013.

Unadjusted 1-year survival improved by 1.0% per year, on average, over the study period (HR: 0.990, 95% CI: 0.987 to 0.993) (table 2 and figure 4). This temporal improvement remained after adjustment for age, sex and deprivation (HR: 0.990, 95% CI, 0.987 to 0.993), cardiac rehabilitation (HR: 0.989, 95% CI: 0.986 to 0.992) and comorbidities and risk factors (HR: 0.993, 95% CI, 0.990 to 0.996). However, the direction of association was reversed after adjustment for PPCI (HR: 1.025, 95% CI: 1.021 to 1.028), and there was no temporal trend after adjusting for pharmacotherapies (HR: 0.998, 95% CI, 0.994 to 1.002). Individual assessments of the pharmacotherapies found that the temporal improvement remained after adjustment for aspirin (HR: 0.988, 95% CI: 0.985 to 0.991), statins (HR: 0.987, 95% CI, 0.984 to 0.989), β-blockers (HR, 0.993; 95% CI: 0.990 to 0.996), ACEi/ARBs (HR: 0.989, 95% CI: 0.986 to 0.992), and only after adjustment of P2Y_12_ inhibitors (HR: 1.035, 95% CI: 1.031 to 1.039) was the direction of association reversed. In the fully adjusted model, the direction of association was reversed (HR: 1.006, 95% CI: 1.001 to 1.011). Comparing a model including only those factors which influenced survival trends with a model including only those factors which did not (model A vs model B, table 3) confirmed that model A variables accounted for improved survival (HR: 1.061, 95% CI: 1.057 to 1.065), whereas the overall temporal trend in survival remained after adjustments made in model B (HR: 0.990, 95% CI: 0.986 to 0.993) (table 3).

**Table 2:** Impact of patient and treatment factors on temporal trends in 6-month and 1-year survival from hospital discharge between 2004 and 2013, for unadjusted and adjusted flexible parametric survival models

Model number	Variables included	Six months	P values	One year	P values
		HR (95% CI)		HR (95% CI)	
Model 1	Year	0.991 (0.988–0.994)	<0.001	0.990 (0.987–0.993)	<0.001
	Year +				
Model 2	Age, sex, IMD	0.991 (0.988 to 0.994)	<0.001	0.990 (0.987 to 0.993)	<0.001
Model 3	PPCI	1.025 (1.021 to 1.029)	<0.001	1.025 (1.021 to 1.028)	<0.001
Model 4	Comorbidities and risk factors	0.994 (0.991 to 0.997)	<0.001	0.993 (0.990 to 0.996)	<0.001
Model 5	Five discharge drugs	0.998 (0.993 to 1.002)	0.404	0.998 (0.994 to 1.002)	0.379
Model 6	Aspirin	0.988 (0.985 to 0.992)	<0.001	0.988 (0.985 to 0.991)	<0.001
Model 7	Statins	0.987 (0.984 to 0.990)	<0.001	0.987 (0.984 to 0.989)	<0.001
Model 8	P2Y_12_ inhibitors	1.040 (1.037 to 1.045)	<0.001	1.035 (1.031 to 1.039)	<0.001
Model 9	ACEi/ARBs	0.991 (0.987 to 0.993)	<0.001	0.989 (0.986 to 0.992)	<0.001
Model 10	β-blockers	0.994 (0.991 to 0.998)	<0.001	0.993 (0.990 to 0.996)	<0.001
Model 11	Cardiac rehabilitation	0.990 (0.987 to 0.993)	<0.001	0.989 (0.986 to 0.992)	<0.001
	Year +age + sex+IMD +				
Model 12	PPCI	1.014 (1.010 to 1.018)	<0.001	1.013 (1.009 to 1.016)	<0.001
Model 13	Comorbidities and risk factors	0.992 (0.989 to 0.996)	<0.001	0.991 (0.988 to 0.994)	<0.001
Model 14	Five discharge drugs	0.993 (0.988 to 0.988)	0.003	0.993 (0.989 to 0.997)	0.001
Model 15	Aspirin	0.987 (0.984 to 0.991)	<0.001	0.987 (0.984 to 0.990)	<0.001
Model 16	Statins	0.968 (0.983 to 0.990)	<0.001	0.986 (0.982 to 0.989)	<0.001
Model 17	P2Y_12_ inhibitors	1.040 (1.035 to 1.044)	<0.001	1.034 (1.030 to 1.038)	<0.001
Model 18	ACEi/ARBs	0.990 (0.986 to 0.993)	<0.001	0.988 (0.985 to 0.992)	<0.001
Model 19	β-blockers	0.994 (0.991 to 0.998)	0.001	0.993 (0.990 to 0.996)	<0.001
Model 20	Cardiac rehabilitation	0.991 (0.987 to 0.994)	<0.001	0.989 (0.986 to 0.992)	<0.001
Model 21	Year +age + sex+IMD + PPCI+comorbidities and risk factors+Aspirin + Statins+P2Y_12_ inhibitors+ACEi/ARBs + β-blockers+cardiac rehabilitation	1.006 (1.001 to 1.011)	0.014	1.006 (1.001 to 1.011)	0.013

ACEi, ACE inhibitor; ARBs, angiotensin receptor blocker; IMD, Index of Multiple Deprivation; PPCI, primary percutaneous coronary intervention.

**Table 3:** Temporal trends by year in overall survival following hospital discharge between 2004 and 2013 for unadjusted and adjusted flexible parametric survival models (comparing model builds for model including only explanatory variables concluded to have an influence improvements in survival trends (model a) with model containing only variables concluded not to influence improvements in survival trends (model B))

	Six months		One year	
	HR (95% CI)	P value	HR (95% CI)	P value
Model A				
Unadjusted yearly time trend	0.991 (0.988 to 0.994)	<0.001	0.990 (0.987 to 0.993)	<0.001
Yearly time trend adjusted for				
P2Y_12_ inhibitors at discharge	1.041 (1.037 to 1.045)	<0.001	1.035 (1.031 to 1.039)	<0.001
P2Y_12_ inhibitors at discharge and PPCI	1.066 (1.061 to 1.071)	<0.001	1.061 (1.057 to 1.065)	<0.001
Model B				
Unadjusted yearly time trend	0.991 (0.988 to 0.994)	<0.001	0.990 (0.987 to 0.993)	<0.001
Yearly time trend adjusted for				
Age, sex and IMD	0.991 (0.988 to 0.994)	<0.001	0.990 (0.987 to 0.993)	<0.001
Age, sex, IMD and pharmacological therapies at discharge (aspirin, statins, β-blockers and ACEi/ARBs)	0.988 (0.984 to 0.992)	<0.001	0.988 (0.985 to 0.991)	<0.001
Age, sex, IMD, pharmacological therapies at discharge (aspirin, statins, β-blockers and ACEi/ARBs) and comorbidities and risk factors	0.990 (0.987 to 0.994)	<0.001	0.990 (0.987 to 0.994)	<0.001
Age, sex, IMD, pharmacological therapies at discharge (aspirin, statins, β-blockers and ACEi/ARBs), comorbidities and risk factors, and cardiac rehabilitation	0.990 (0.986 to 0.993)	<0.001	0.990 (0.986 to 0.993)	<0.001

ACEi, ACE inhibitor; ARBs, angiotensin receptor blocker; IMD, Index of Multiple Deprivation; PPCI, primary percutaneous coronary intervention.

### Six-month survival

Unadjusted 6-month survival improved by 0.9% per year, on average, over the study period (HR: 0.991, 95% CI: 0.988 to 0.994) (table 2 and figure 4). This temporal improvement remained after adjustment for age, sex and deprivation (HR: 0.991, 95% CI: 0.988 to 0.994), cardiac rehabilitation (HR: 0.990, 95% CI: 0.987 to 0.993) and comorbidities and risk factors (HR: 0.994, 95% CI: 0.991 to 0.997). However, the direction of association was reversed after adjustment for PPCI (HR: 1.025, 95% CI: 1.021 to 1.029) and there was no temporal trend after adjusting for pharmacotherapies (HR: 0.998, 95% CI: 0.993 to 1.002). Individual assessments of the pharmacotherapies showed that the temporal improvement remained after adjusting for aspirin (HR: 0.988, 95% CI: 0.985 to 0.992), statins (HR: 0.987, 95% CI: 0.984 to 0.990), β-blockers (HR: 0.994, 95% CI: 0.991 to 0.998), ACEi/ARBs (HR: 0.991, 95% CI: 0.987 to 0.993). Only after adjustment for receipt of P2Y_12_ inhibitors was the direction of association reversed (HR: 1.040, 95% CI: 1.037 to 1.045). In the fully adjusted model, the direction of association was reversed (HR: 1.006, 95% CI: 1.001 to 1.011). Comparing a model including only the factors that influenced survival trends with a model including only those factors that did not (model A vs model B, table 3) confirmed that model A variables accounted for improved survival (HR 1.066, 95% CI 1.061 to 1.071), whereas the overall temporal trend in survival remained after adjustments made in model B (HR: 0.990, 95% CI: 0.986 to 0.993) (table 3). Given that the number of people with prior use of statin at presentation of STEMI declined over the study period, we conducted a sensitivity analyses stratified according to prior statin use in which our findings were substantiated (online supplementary eTables 4 and 5).

### Causal mediation analysis

The uptake of PPCI explained 13.2% (95% CI: 9.2% to 21.9%; average across the 10 imputed datasets) of the 1-year and 16.8% (95% CI: 10.8% to 31.6%) of the 6-month survival improvements (online supplementary eTables 6 and 7). The increased prescription of P2Y_12_ inhibitors at hospital discharge explained 5.3% (95% CI: 3.6% to 8.8%) of the improvement at 6 months, but not at 1 year (online supplementary eTables 6 and 7). A sensitivity analysis including in-hospital deaths was carried out and the results were consistent with the main findings (online supplementary eTables 8 and 9).

## Discussion

In this national cohort study of the management and outcome of nearly a quarter of a million patients hospitalised with STEMI, we found that temporal improvements in 1-year survival between 2004 and 2013 were associated with the uptake of PPCI and increased prescription of P2Y_12_ inhibitors at hospital discharge. Similar findings were observed for temporal improvements in 6-month survival. At the end of the study, the use of guideline-indicated therapies for STEMI was high, such that over three-quarters of patients with STEMI received PPCI, and the prescription of evidence-based pharmacotherapies reached more than 95%. While the majority of treatment and patient-related factors studied were significantly associated with 6-month and 1-year survival, it was only temporal trends in PPCI and P2Y_12_ inhibitors at hospital discharge that significantly explained the temporal trend in survival.

The association between guideline recommended care and improved survival after myocardial infarction is recognised and supported by robust evidence from randomised clinical trials.^10^ For STEMI, PPCI has been shown to be associated with a decline in mortality,^11–13^ as has the use of antithrombotic and secondary prevention medications.^3 4 14 15^ While others have investigated whether these or other factors are associated with the decline in mortality over time,^3–5^ to our knowledge this is the largest study to date and first investigation using causal medication analysis to determine the relative contribution of factors on survival improvements in STEMI.

Our findings support and extend the existing literature.^3–5^ A recent study of 105 674 patients with STEMI recorded in the Swedish Web-System for Enhancement and Development of Evidence-Based Care in Heart Disease Evaluated According to Recommended Therapies registry found that changes in reperfusion and PPCI were each associated with improved in-hospital outcomes, whereas an increase in the prescription of discharge medications was associated with improved outcomes at 1 year. Our study builds on this, finding that the nationwide introduction of PPCI explained over one-tenth of the improvements in 1-year survival for STEMI.

We found no significant mediation effect for improvements in 1-year survival by increased prescription of P2Y_12_ inhibitors at hospital discharge. By contrast, a significant effect was identified for survival improvements at 6 months. Debate remains as to the most appropriate duration of dual antiplatelet therapy following myocardial infarction^16–18^ and in the UK variation exists as to whether this is from 3 to 12 months or longer.^19^ Thus, it is possible that persistence with therapy influenced the duration of the causal effect. Alternatively, it may be that a second antiplatelet agent following STEMI has limited benefit for longer-term all-cause mortality.^18^

The finding that the uptake of P2Y_12_ inhibitors and PPCI significantly explained the temporal trends in survival is not surprising. P2Y_12_ inhibitors are indicated with a Class I recommendation and Level A evidence^10^ for all STEMI before, or at the time of PPCI. As such, one would expect a moderating relationship between PPCI and P2Y_12_ inhibitors. Yet, the uptake of PPCI and increased prescription of P2Y_12_ inhibitors did not explain all of the improvements in survival. Earlier research found that organisational factors, as well as national and local infrastructure are key elements in the delivery of a STEMI service and, therefore, clinical outcomes.^2^

This study has wider implications for international cardiovascular health. In line with the WHO Global Action Plan for non-communicable disease, we have identified factors including and beyond PPCI that are associated with improved survival over time for patients with STEMI—therefore helping identify where in a healthcare system the provision of essential treatments is required to reduce premature death. Furthermore, our results may be extrapolated to other developed and developing countries which lag behind Northern Europe and North America in their provision of care and where greater gains in cardiovascular health maybe realised.^20 21^

MINAP is the largest whole-country, single-health-system, prospective observational cohort of the quality of care and clinical outcomes for ACS. It is designed to be representative of the management of ACS and has standardised criteria for defining case mix and treatments. To our knowledge, this study is the first to quantify the relative contribution mediators of temporal improvements in survival for STEMI. However, our study has limitations. (1) We were reliant on the accurate recording of data in MINAP. (2) MINAP collects the majority, but not all cases of STEMI in England and Wales. (3) Missing data could have biassed our estimates; to mitigate this we studied the nature of the missing data and used imputation algorithms. (4) Other factors beyond the hospital stay (such as drug adherence and compliance, and primary care visits) may have influenced temporal changes in survival. (5) We studied all-cause mortality, when non-cardiovascular deaths may not be attributable to temporal improvements in STEMI care.^22^ (6) Given that the determined mediators did not fully explain the survival improvements implies unmeasured mediators exist out with MINAP data fields. (7) MINAP lacks information on temporal change in stent platforms, that is, shift from bare metal stents to first-generation and second-generation drug eluting stents. (8) Door-to-balloon and total ischaemic times were not investigated as potential mediators due to poor recording of this information within our MINAP extract.

## Conclusion

Among 232 353 patients hospitalised with STEMI in England and Wales, improvements in all-cause mortality between 2004 and 2013 were significantly associated with, and explained by, the national uptake of PPCI and increased prescription of P2Y_12_ inhibitors, and not entirely related to changes in comorbidities or increased use of other pharmacological therapies.

**Key messages**

**What was already known on this subject?**

- Temporal improvements in survival following ST-elevation Myocardial Infarction (STEMI) have been observed and the improvements have been attributed to adoption of new health technologies such as primary percutaneous coronary intervention (PPCI) as well as the availability of novel pharmacotherapies. However, the relative contributions of temporal improvements in treatments and patient characteristics on improved STEMI survival are unknown.

**What might this study add?**

- While the majority of treatment and patient-related factors studied were significantly associated with STEMI survival, it was only temporal improvements in P2Y_12_ inhibitors prescription and national uptake of PPCI that significantly explained the temporal trend in STEMI survival observed between 2004 and 2013.

**How might this impact on clinical practice?**

- Identifying factors associated with improved survival over time for patients with STEMI helps identify where in healthcare systems the provision of essential treatments is required to reduce premature death from cardiovascular disease.
