# Supplementary material for: A nationwide causal mediation analysis of survival following ST-elevation myocardial infarction
Source: Heart. 2019 Nov 15;106(10):765–71. doi: 10.1136/heartjnl-2019-315760 (PMC7229897; doi:10.1136/heartjnl-2019-315760)
Supplement: Supplementary data [file heartjnl-2019-315760supp001.pdf]

SUPPLEMENTAL MATERIAL

A nationwide causal mediation analysis of survival following ST-elevation Myocardial Infarction

Authors

Tatendashe B Dondo (PhD), Marlous Hall (PhD), Theresa Munyombwe (PhD), Chris Wilkinson (MRCP), Mohammad E Yadegarfar (PhD), Adam D Timmis (FRCP), Phillip D Batin (FRCP), Tomas Jernberg (FRCP), Keith AA Fox (FRCP), Chris P Gale (FRCP).

Dondo TB, *et al. Heart* 2020; 106:765–771. doi: 10.1136/heartjnl-2019-315760

Section 1

eTable 1. Mapping of care interventions for the management of STEMI to MINAP data fields.

**Care intervention**	**Eligibility Criteria**	**Opportunity Received**
**Aspirin**		
At Discharge	If discharged on aspirin (4.08) is not =2 (contraindicated), 3 (patient declined treatment), 4 (not applicable) or 8 (not indicated), as recorded in MINAP.	Discharged on aspirin (4.08)=1 (yes)
**$P2Y_{12}$ inhibitor**		
At Discharge	If discharged on thienopyridine (4.27) is not =2 (contraindicated), 3 (patient declined treatment),4 (not applicable) or 8 (not indicated) Or if discharged on ticagrelor is not =2 (contraindicated),3 (patient declined treatment),4 (not applicable),8 (not indicated) , as recorded in MINAP.	If discharged on thienopyridine=1 (yes) or if discharged on ticagrelor=1 (yes)
**β-Blocker**		

At Discharge	If discharged on β blockers (4.05) is not =2 (contraindicated), 3 (patient declined treatment), 4 (not applicable) or 8 (not indicated), as recorded in MINAP.	If discharged on beta blocker(4.05)=1 (yes)
**ACEi/ARBs**		
At Discharge	If discharged on ACEi/ARB (3.32/4.06) is not =2 (contraindicated), 3 (patient declined treatment), 4 (not applicable) or 8 (not indicated), as recorded in MINAP.	If ACEi/ARB (3.32)=1 (yes) OR if discharged ACEi/ARB (4.06)=1 (yes)
**Statin**		
At Discharge	If discharged on statin (4.07) is not =2 (contraindicated), 3 (patient declined treatment), 4 (not applicable) or 8 (not indicated), as recorded in MINAP.	If statin (4.07)=1 (yes)
**Reperfusion strategy**		
PPCI	If reason no reperfusion (3.08) was given is not= 1 (ineligible ECG), 2 (too late), 3 (risk of haemorrhage), 4 (uncontrolled hypertension), 5	If initial_reperfusion (3.39)= 2 (PPCI in house), 3 (referred for consideration for PPCI elsewhere), 4 (PPCI already

Dondo TB, *et al. Heart* 2020; 106:765–771. doi: 10.1136/heartjnl-2019-315760

	(administrative failure), 6 (elective decision), 7 (patient refused treatment) or 8 (other).	was performed at the interventional hospital)
Thrombolysis	If reason no reperfusion (3.08) was given is not = 1 (ineligible ECG), 2 (too late), 3 (risk of haemorrhage), 4 (uncontrolled hypertension), 5 (administrative failure), 6 (elective decision), 7 (patient refused treatment) or 8 (other).	If lytic (3.36) =1 (Streptokinase), 2 (Alteplase), 3 (Reteplase) or 4 (Tenecteplase) or initial_reperfusion (3.39) = 1 (thrombolytic treatment).
**Referral for Cardiac Rehabilitation**	All patients eligible unless not indicated<br><br>If cardiac rehabilitation (4.09) is not =3 (patient declined treatment) or 8 (not indicated), as recorded in MINAP.	If cardiac rehabilitation (4.09)=1 (yes)

Abbreviations: ACEi – angiotensin-converting enzyme inhibitor; ARBs – Angiotensin receptor blocker; ECG – electrocardiogram; PPCI – primary percutaneous coronary intervention; MINAP- Myocardial Ischaemia National Audit Project.

Dondo TB, *et al. Heart* 2020; 106:765–771. doi: 10.1136/heartjnl-2019-315760

Section 2

Flexible parametric survival modelling

eTable 2. Choice of scale and degrees of freedom for the flexible parametric survival model determined by minimisation of the Akaike's Information Criterion and Bayes Information Criterion.

	**AIC and BIC Ranges Over 10 Imputed Data Sets**							
**df**	**Normal**		**Theta**		**Odds**		**Hazard**	
	AIC	BIC	AIC	BIC	AIC	BIC	AIC	BIC
1	44583.12-59707.10	44766.77-59892.90	44679.12-59838.09	44869.10-60030.52	44763.57-59949.11	44947.22-60134.91	44802.32-60001.58	44985.97-60187.38
2	44362.39-59388.40	44552.38-59580.83	44447.19-59497.81	44643.50-59696.88	44469.44-59520.88	44659.42-59713.32	44491.38-59548.08	44681.36-59740.51
3	44356.42-59383.37	44552.74-59582.44	44441.39-59491.35	44644.04-59697.06	44463.05-59513.45	44659.36-59712.52	44484.70-59540.26	44681.01-59739.33

Dondo TB, *et al. Heart* 2020; 106:765–771. doi: 10.1136/heartjnl-2019-315760

4	44339.93-59348.65	44542.58-59554.36	44422.30-59451.52	44631.28-59663.86	44442.99-59471.96	44645.64-59677.67	44464.42-59498.20	44667.07-59703.91
5	**44324.97-59322.65**	**44533.95-59534.99**	44405.02-59421.74	44620.33-59640.72	44425.09-59441.33	44634.07-59653.68	44446.35-59467.28	44655.33-59679.62

Abbreviations: AIC- Akaike's Information Criterion; BIC- Bayes Information Criterion; df-degrees of freedom.

Dondo TB, *et al. Heart* 2020; 106:765–771. doi: 10.1136/heartjnl-2019-315760

Section 2

Mediation analysis

A mediation analysis was conducted to determine the proportion of temporal improvements in survival that were mediated by use of PPCI and prescription of $P2Y_{12}$ inhibitors at hospital discharge (eFigure 1). Details on the method and R package used for the analyses have been described elsewhere.[1, 2] The mediation analysis was undertaken on complete cases (n=137,111) and across each of the ten imputed datasets separately as methods to pool the estimates to the best of our knowledge have not been established.

eFigure 1. A path diagram indicating the use of PPCI and prescription of $P2Y_{12}$ inhibitors at hospital discharge as potential mediators for the temporal trend in survival.

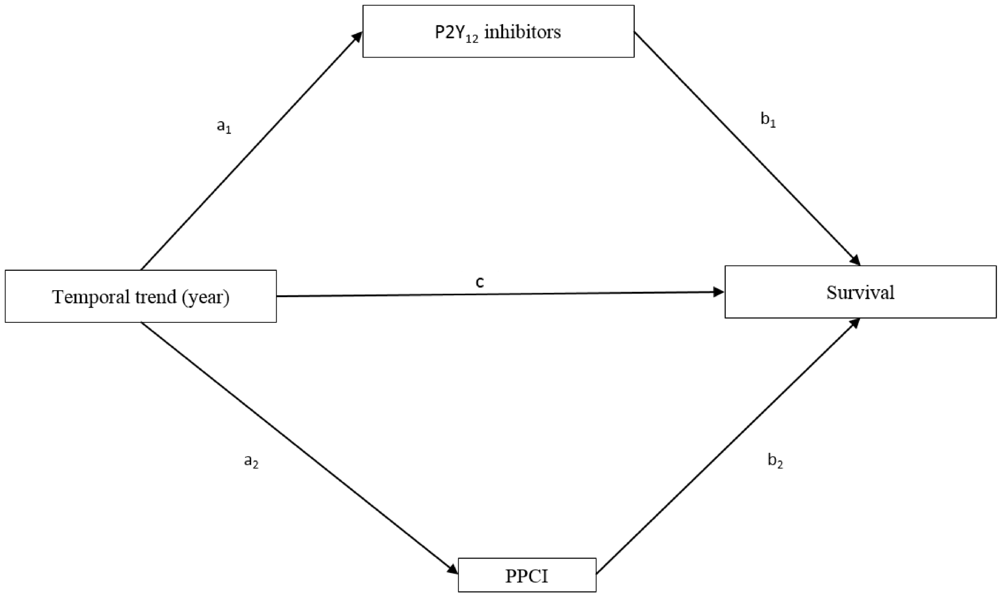

Since two potential mediators were determined in the primary analysis (eFigure 1), two independent single mediator models for the hypothesised mediators (PPCI and prescription of

P2Y12 inhibitors at hospital discharge) were fitted. For each model average total effects (ATE), average causal mediation effect (ACME), average direct effect (ADE), and the proportion mediated by the hypothesised mediators were determined. Average direct effects (ADE) are defined as the intervention effects on outcome after excluding the mediator effects (represented by path c in eFigure 1), while the ACME is defined as the intervention effect on the outcome via the mediator (represented by paths a_1, a_2, b_1 and b_2 in eFigure 1).[2, 3] The ATE is the sum of ADE and ACME, which is defined as the total intervention effects on the outcome.[2, 3] The proportion mediated is the fraction of ATE that is explained by ACME.[2, 3] The ACME and ADE are estimated under the potential outcomes framework whereby the impact of the mediator on the outcome is quantified comparing impact on outcomes if everyone in the population received treatment/mediating variable vs. if no one in the population received treatment/mediating variable.[3] The potential outcomes come into play in the sense that not everyone has an observed outcome if shifted to the different treatment groups other than their observed treatment group, thus the employment of counterfactual outcomes (potential outcomes).[3]

For each of the mediators, two regression models were fitted, i.e. the mediator model (to quantify ACME) and the outcome model (to quantify ATE). In the mediator model, year of admission was the independent variable and the hypothesised mediator as the dependent variable. However, in order to infer causal inference the mediation analysis's main assumption: the sequential ignorability assumption must not be violated. The sequential ignorability assumption assumes that the mediator is effectively randomly assigned given pre-intervention covariates and the randomised treatment.[4] Given that in observational studies, potential bias may arise at ether treatment assignment or mediator stage [4], the mediator models were adjusted for pre-intervention covariates. The pre-intervention covariates included: age, sex, deprivation (Index of Multiple Deprivation score), diabetes,

Dondo TB, *et al. Heart* 2020; 106:765–771. doi: 10.1136/heartjnl-2019-315760

hypercholesterolaemia, hypertension, smoking status, family history of coronary heart disease, chronic obstructive pulmonary disease, cerebrovascular disease, peripheral vascular disease, previous history of AMI, previous history of angina, previous coronary revascularisation, previous coronary artery bypass graft. This was done to ensure that the mediating variables were as good as randomised conditional on the pre-intervention covariates adjusted for in the mediator models. Thus allowing for interpretation of paths b_1 and b_2 as causal because individuals within each treatment group attaining different levels of the mediator would be similar. For the outcome models, we used a Poisson regression modelling framework with log survival time as the offset (in the absence of software packages available to fit flexible parametric survival models for mediation analysis).

The outcome models were adjusted for patient demographics (sex, deprivation (index of multiple deprivation score)), cardiovascular risk factors (diabetes, hypercholesterolaemia, hypertension, smoking status, chronic obstructive pulmonary disease (COPD), family history of coronary heart disease, previous history of AMI, previous history of angina, previous coronary revascularisation, previous coronary artery bypass graft), cardiovascular history (cerebrovascular disease, peripheral vascular disease), discharge medications (statins, aspirin, angiotensin converting enzyme inhibitors (ACEi)/angiotensin receptor blockers (ARB)) and cardiac rehabilitation. Cardiac rehabilitation was added only to the outcome models because it is a post intervention variable and therefore could not be included in the mediator model as a pre-intervention covariate. To take into account the mediated effect of the other mediator, they were adjusted for in each of the outcome models in turn. However, this can prove a limitation if the indirect effects of the mediators have an opposite signs, it can result to the indirect effects either cancelling each other out if the effect sizes are the same (thus resulting to no effect being mediated) or an underestimation of the proportion mediated if the indirect effects are not the same size.[5] In this analysis, the indirect effects were assumed to have the

same sign as all the mediators had the same impact on survival of the STEMI patients. This assumption was confirmed to hold true as none of the total effects from the individual models for each of the mediators were found to be zero (eTable 6 and eTable 7). The mediate function was used to estimate ATE, ACME, ADE and the proportion mediated by determined mediators.[2]

The modelling approach was undertaken for the both the primary and secondary outcomes, i.e. one year and six months survival, respectively. Results of the findings are given in eTable 6 and eTable 7.

Dondo TB, *et al. Heart* 2020; 106:765–771. doi: 10.1136/heartjnl-2019-315760

Section 3

Multiple Imputation

Multiple imputations by chained equations (MICE)[6] were used to create 10 imputed datasets for missing data for all components of the GRACE risk score and other patient demographic variables. A default imputation (missing data default imputed to “NO”) strategy based on clinical expert opinion was implemented for cardiovascular history, cardiovascular risk factors, and categorical treatment variables.[6, 7] The imputation models were based on previous work. The imputation model used is defined in detail in eTable 3. Predictive mean matching was used for continuous variables with nonlinear associations.

Dondo TB, *et al. Heart* 2020; 106:765–771. doi: 10.1136/heartjnl-2019-315760

eTable 3. Imputation Strategy

**Variable**	**Variable Type**	**Missing (%)**	**Imputation method**
Cardiac arrest	Binary	7.9	Logistic regression
Uncensored peak troponin measurement in ng/ml	Continuous	22.0	Predictive mean matching
Age	Continuous	0.1	Predictive mean matching
Systolic blood pressure	Continuous	18.5	Predictive mean matching
Heart rate	Continuous	18.1	Predictive mean matching
Loop diuretic used	Binary	20.4	Logistic regression
Creatinine level	Continuous	41.2	Predictive mean matching
Ethnicity	Categorical	11.8	Polytomous regression
Sex	Binary	0.3	Logistic regression
Index of multiple deprivation score	Continuous	7.6	Predictive mean matching
Derived identification	Continuous	0	Predictor/ Auxiliary /Partially Observed
Arrival year	Continuous	0	Predictor/ Auxiliary /Partially Observed
Nelson-Aalen survival estimate	Continuous	0	Predictor/ Auxiliary /Partially Observed

Dondo TB, *et al. Heart* 2020; 106:765–771. doi: 10.1136/heartjnl-2019-315760

**Variable**	**Variable Type**	**Missing (%)**	**Imputation method**
Censoring indicator	Binary	0	Predictor/ Auxiliary /Partially Observed
Hypercholesterolaemia	Binary	12.9	Predictor/ Auxiliary and Default imputed
Previous hypertension	Binary	9.5	Predictor/ Auxiliary and Default imputed
Previous myocardial infarction	Binary	9.1	Predictor/ Auxiliary and Default imputed
Previous angina	Binary	10.4	Predictor/ Auxiliary and Default imputed
Previous PCI	Binary	11.0	Predictor/ Auxiliary and Default imputed
Previous CABG	Binary	10.8	Predictor/ Auxiliary and Default imputed
Peripheral vascular disease	Binary	12.6	Predictor/ Auxiliary and Default imputed
Cerebrovascular disease	Binary	11.8	Predictor/ Auxiliary and Default imputed
Chronic obstructive pulmonary disease/Asthma	Binary	12.5	Predictor/ Auxiliary and Default imputed

Variable	Variable Type	Missing (%)	Imputation method
Smoker ever	Binary	9.3	Logistic regression
Diabetes	Binary	7.1	Predictor/ Auxiliary and Default imputed
Family history of chronic heart disease	Binary	31.1	Predictor/ Auxiliary and Default imputed
Care by Cardiologist	Binary	33.1	Predictor/ Auxiliary and Default imputed
Chronic renal failure	Binary	11.8	Predictor/ Auxiliary and Default imputed
Congestive cardiac failure	Binary	11.5	Predictor/ Auxiliary and Default imputed
Electrocardiogram appearance	Categorical	3.4	Polytomous regression
Preadmission medication			
Aspirin	Categorical	0	Predictor/ Auxiliary and Default imputed
β-blocker	Categorical	28.1	Predictor/ Auxiliary and Default imputed
Statin	Categorical	25.3	Predictor/ Auxiliary and Default imputed

Dondo TB, *et al. Heart* 2020; 106:765–771. doi: 10.1136/heartjnl-2019-315760

**Variable**	**Variable Type**	**Missing (%)**	**Imputation method**
ACEi or ARBs	Categorical	28.3	Predictor/ Auxiliary and Default imputed
$P2Y_{12}$ inhibitor	Categorical	60.0	Predictor/ Auxiliary and Default imputed
Warfarin	Categorical	20.2	Predictor/ Auxiliary and Default imputed
Discharge medication			
Aspirin	Categorical	8.4	Polytomous regression
$P2Y_{12}$ inhibitors	Categorical	52.3	Polytomous regression
ACEi/ARBs	Categorical	9.2	Polytomous regression
Statin	Categorical	8.6	Polytomous regression
β blockers	Categorical	16.2	Polytomous regression
Aldosterone antagonist	Categorical	56.7	Polytomous regression
Enzyme elevation	Binary	9.7	Predictor/ Auxiliary variable
Admission diagnosis	Categorical	0	Predictor/ Auxiliary variable
Admitting consultant	Binary	5.2	Predictor/ Auxiliary variable

Dondo TB, *et al. Heart* 2020; 106:765–771. doi: 10.1136/heartjnl-2019-315760

**Variable**	**Variable Type**	**Missing (%)**	**Imputation method**
Serum cholesterol	Continuous	26.1	Predictor/ Auxiliary variable
Coronary angiography	Categorical	12.6	Polytomous regression
Coronary intervention	Categorical	19.4	Polytomous regression
Cardiac rehabilitation	Categorical	11.4	Polytomous regression

Abbreviations: ARB – Angiotensin receptor blocker; ACEi – angiotensin-converting enzyme inhibitor; CABG – coronary artery bypass grafting; COPD – chronic obstructive pulmonary disease; PCI – percutaneous coronary intervention.

Dondo TB, *et al. Heart* 2020; 106:765–771. doi: 10.1136/heartjnl-2019-315760

Section 4

eTable 4. Impact of patient and treatment factors on temporal trends in six months and one year survival between 2004 and 2013, for unadjusted and adjusted flexible parametric survival models (**excluding patients with prior statin therapy, N= 119,367**).

		Six months		One year	
**Model number**	**Variables included**	**Hazard ratio (95% CI)**	**P-value**	**Hazard ratio (95% CI)**	**P-value**
Model 1	Year	0.988 (0.984-0.991)	<0.001	0.986 (0.982-0.989)	<0.001
	Year +				
Model 2	Age, sex, IMD	0.990 (0.987-0.994)	<0.001	0.989 (0.985-0.992)	<0.001
Model 3	PPCI	1.019 (1.015-1.023)	<0.001	1.017 (1.013-1.021)	<0.001
Model 4	Comorbidities and risk factors	0.999 (0.995-1.003)	0.531	0.998 (0.995-1.002)	0.354
Model 5	Five discharge drugs	0.990 (0.985-0.995)	<0.001	0.989 (0.985-0.994)	<0.001
Model 6	Aspirin	0.983 (0.979-0.987)	<0.001	0.982 (0.979-0.985)	<0.001
Model 7	Statins	0.981 (0.977-0.985)	<0.001	0.980 (0.977-0.983)	<0.001
Model 8	$P2Y_{12}$ inhibitors	1.034 (1.030-1.039)	<0.001	1.027 (1.023-1.032)	<0.001
Model 9	ACEi/ARBs	0.985 (0.982-0.989)	<0.001	0.983 (0.980-0.987)	<0.001
Model 10	β-blockers	0.989 (0.986-0.993)	<0.001	0.987 (0.984-0.990)	<0.001
Model 11	Cardiac rehabilitation	0.986 (0.982-0.990)	<0.001	0.984 (0.981-0.987)	<0.001
	Year + age + sex + IMD +				
Model 12	PPCI	1.012 (1.007-1.016)	<0.001	1.010 (1.006-1.014)	<0.001
Model 13	Comorbidities and risk factors	0.996 (0.992-0.999)	0.037	0.995 (0.992-0.999)	0.008
Model 14	Five discharge drugs	0.987 (0.982-0.993)	<0.001	0.988 (0.983-0.992)	<0.001
Model 15	Aspirin	0.985 (0.980-0.989)	<0.001	0.984 (0.980-0.987)	<0.001
Model 16	Statins	0.983 (0.979-0.986)	<0.001	0.981 (0.978-0.985)	<0.001
Model 17	$P2Y_{12}$ inhibitors	1.036 (1.031-1.041)	<0.001	1.030 (1.025-1.034)	<0.001
Model 18	ACEi/ARBs	0.987 (0.983-0.991)	<0.001	0.986 (0.982-0.989)	<0.001

Dondo TB, *et al. Heart* 2020; 106:765–771. doi: 10.1136/heartjnl-2019-315760

		Six months		One year	
**Model number**	**Variables included**	**Hazard ratio (95% CI)**	**P-value**	**Hazard ratio (95% CI)**	**P-value**
Model 19	β-blockers	0.992 (0.988-0.996)	<0.001	0.990 (0.987-0.993)	<0.001
Model 20	Cardiac rehabilitation	0.989 (0.985-0.993)	<0.001	0.987 (0.984-0.991)	<0.001
Model 21	Year + age + sex + IMD + PPCI + Comorbidities and risk factors + Aspirin + Statins + $P2Y_{12}$ inhibitors + ACEi/ARBs + β-blockers + Cardiac rehabilitation	1.005 (1.000-1.011)	0.069	1.006 (1.001-1.012)	0.020

Abbreviations: ACEi – angiotensin-converting enzyme inhibitor; ARBs – Angiotensin receptor blocker; IMD – index of multiple deprivation and PPCI – primary percutaneous coronary intervention.

Dondo TB, *et al. Heart* 2020; 106:765–771. doi: 10.1136/heartjnl-2019-315760

eTable 5. Impact of patient and treatment factors on temporal trends in six months and one year survival between 2004 and 2013, for unadjusted and adjusted flexible parametric survival models (**restricted to patients with prior statin therapy only, N=54,151**).

		**Six months**		**One year**	
**Model number**	**Variables included**	**Hazard ratio (95% CI)**	**P-value**	**Hazard ratio (95% CI)**	**P-value**
Model 1	Year	0.994 (0.987-1.001)	0.087	0.993 (0.986-0.999)	0.021
	Year +				
Model 2	Age, sex, IMD			0.986 (0.979-0.993)	<0.001
Model 3	PPCI			1.029 (1.022-1.036)	<0.001
Model 4	Comorbidities and risk factors			0.996 (0.989-1.002)	0.182
Model 5	Five discharge drugs			1.017 (1.008-1.026)	<0.001
Model 6	Aspirin			0.997 (0.991-1.004)	0.433
Model 7	Statins			0.996 (0.990-1.002)	0.213
Model 8	$P2Y_{12}$ inhibitors			1.050 (1.041-1.058)	<0.001
Model 9	ACEi/ARBs			0.998 (0.992-1.004)	0.528
Model 10	β-blockers			1.001 (0.995-1.008)	0.614
Model 11	Cardiac rehabilitation			0.995 (0.989-1.001)	0.122
	Year + age + sex + IMD +				
Model 12	PPCI			1.011 (1.004-1.019)	0.004
Model 13	Comorbidities and risk factors			0.988 (0.981-0.994)	<0.001
Model 14	Five discharge drugs			1.002 (0.992-1.011)	0.725
Model 15	Aspirin			0.990 (0.983-0.997)	0.003
Model 16	Statins			0.988 (0.981-0.995)	0.001
Model 17	$P2Y_{12}$ inhibitors			1.039 (1.030-1.048)	<0.001
Model 18	ACEi/ARBs			0.990 (0.983-0.997)	0.003
Model 19	β-blockers			0.994 (0.987-1.001)	0.073
Model 20	Cardiac rehabilitation			0.988 (0.981-0.995)	<0.001

Dondo TB, *et al. Heart* 2020; 106:765–771. doi: 10.1136/heartjnl-2019-315760

		Six months		One year	
**Model number**	**Variables included**	**Hazard ratio (95% CI)**	**P-value**	**Hazard ratio (95% CI)**	**P-value**
Model 21	Year + age + sex + IMD + PPCI + Comorbidities and risk factors + Aspirin + Statins + $P2Y_{12}$ inhibitors + ACEi/ARBs + β-blockers + Cardiac rehabilitation			1.009 (1.000-1.019)	0.057

Abbreviations: ACEi – angiotensin-converting enzyme inhibitor; ARBs – Angiotensin receptor blocker; IMD – index of multiple deprivation and PPCI – primary percutaneous coronary intervention

Dondo TB, *et al. Heart* 2020; 106:765–771. doi: 10.1136/heartjnl-2019-315760

Section 5

eTable 6. Mediation analysis modelling for one year survival, by imputation dataset.

Imputation dataset	Analysis	Intervention-mediator effect (path a_1 and a_2)	Mediator-outcome effect (path b_1 and b_2)	ADE	ACME	Proportion mediated (%)	P value
0*	$P2Y_{12}$ inhibitors	-0.07 (-0.08 to -0.05)	-0.16 (-0.27 to -0.04)	7.95×10^{-5} (-4.64×10^{-5} to 1.71×10^{-4})	5.41×10^{-6} (-4.26×10^{-7} to 1.36×10^{-5})	-	-
	Introduction of PPCI	0.32 (0.32 to 0.34)	-0.37 (-0.43 to -0.30)	1.98×10^{-4} (8.98×10^{-5} to 2.69×10^{-4})	-5.07×10^{-5} (-6.62×10^{-5} to -3.80×10^{-5})	-32.9 (-100 to -17.1)	0.030
1	$P2Y_{12}$ inhibitors	0.79 (0.78 to 0.79)	-0.23 (-0.29 to -0.19)	-4.50×10^{-6} (-6.77×10^{-5} to 6.1210^{-5})	-9.26×10^{-6} (-1.12×10^{-5} to -7.25×10^{-6})	-	-
	Introduction of PPCI	0.63 (0.62 to 0.63)	-0.40 (-0.44 to -0.36)	1.70×10^{-4} (1.17×10^{-4} to 2.22×10^{-4})	-1.98×10^{-5} (-2.29×10^{-5} to -1.67×10^{-5})	-13.1 (-21.7 to -9.2)	<0.001

Dondo TB, *et al. Heart* 2020; 106:765–771. doi: 10.1136/heartjnl-2019-315760

**Imputation dataset**	**Analysis**	**Intervention-mediator effect (path a_1 and a_2)**	**Mediator-outcome effect (path b_1 and b_2)**	**ADE**	**ACME**	**Proportion mediated (%)**	**P value**
2	$P2Y_{12}$ inhibitors	0.79 (0.78 to 0.79)	-0.24 (-0.29 to -0.19)	-4.49×10^{-6} (-6.78×10^{-5} to 6.11×10^{-5})	-9.27×10^{-6} (-1.13×10^{-5} to -7.17×10^{-6})	-	-
	Introduction of PPCI	0.63 (0.62 to 0.63)	-0.40 (-0.44 to -0.36)	1.70×10^{-4} (1.17×10^{-4} to 2.22×10^{-4})	-1.97×10^{-5} (-2.27×10^{-5} to -1.67×10^{-5})	-13.1 (-21.6 to -9.2)	<0.001
3	$P2Y_{12}$ inhibitors	0.79 (0.78 to 0.79)	-0.24 (-0.29 to -0.19)	-7.11×10^{-6} (-7.09×10^{-5} to 5.86×10^{-5})	-9.22×10^{-6} (-1.12×10^{-5} to -7.11×10^{-6})	-	-
	Introduction of PPCI	0.63 (0.62 to 0.63)	-0.40 (-0.44 to -0.36)	1.68×10^{-4} (1.15×10^{-4} to 2.20×10^{-4})	-1.97×10^{-5} (-2.27×10^{-5} to -1.68×10^{-5})	-13.4 (-22.2 to -9.3)	<0.001
4	$P2Y_{12}$ inhibitors	0.79 (0.78 to 0.79)	-0.24 (-0.29 to -0.19)	-1.03×10^{-5} (-7.40×10^{-5} to 5.57×10^{-5})	-9.20×10^{-6} (-1.12×10^{-5} to -7.18×10^{-6})	-	-

Dondo TB, *et al. Heart* 2020; 106:765–771. doi: 10.1136/heartjnl-2019-315760

**Imputation dataset**	**Analysis**	**Intervention-mediator effect (path a_1 and a_2)**	**Mediator-outcome effect (path b_1 and b_2)**	**ADE**	**ACME**	**Proportion mediated (%)**	**P value**
	Introduction of PPCI	0.63 (0.62 to 0.63)	-0.40 (-0.44 to -0.36)	1.66×10^{-4} (1.12×10^{-4} to 2.18×10^{-4})	-1.98×10^{-5} (-2.28×10^{-5} to -1.68×10^{-5})	-13.6 (-22.9 to -9.4)	<0.001
5	$P2Y_{12}$ inhibitors	0.79 (0.78 to 0.79)	-0.24 (-0.29 to -0.19)	-5.23×10^{-6} (-6.87×10^{-5} to 6.03×10^{-5})	-9.28×10^{-6} (-1.14×10^{-5} to -7.25×10^{-6})	-	-
	Introduction of PPCI	0.63 (0.62 to 0.63)	-0.40 (-0.44 to -0.35)	1.69×10^{-4} (1.16×10^{-4} to 2.21×10^{-4})	-1.97×10^{-5} (-2.27×10^{-5} to -1.68×10^{-5})	-13.2 (-21.7 to -9.2)	<0.001
6	$P2Y_{12}$ inhibitors	0.79 (0.78 to 0.79)	-0.24 (-0.29 to -0.19)	-6.98×10^{-6} (-7.04×10^{-5} to 5.88×10^{-5})	-9.22×10^{-6} (-1.14×10^{-5} to -7.17×10^{-6})	-	-
	Introduction of PPCI	0.63 (0.62 to 0.63)	-0.40 (-0.44 to -0.36)	1.67×10^{-4} (1.14×10^{-4} to 2.19×10^{-4})	-1.97×10^{-5} (-2.27×10^{-5} to -1.67×10^{-5})	-13.4 (-22.3 to -9.2)	<0.001

Dondo TB, *et al. Heart* 2020; 106:765–771. doi: 10.1136/heartjnl-2019-315760

Imputation dataset	Analysis	Intervention-mediator effect (path a_1 and a_2)	Mediator-outcome effect (path b_1 and b_2)	ADE	ACME	Proportion mediated (%)	P value
7	$P2Y_{12}$ inhibitors	0.79 (0.78 to 0.79)	-0.24 (-0.29 to -0.19)	-6.09×10^{-6} (-6.95×10^{-5} to 5.97×10^{-5})	-9.25×10^{-6} (-1.13×10^{-5} to -7.23×10^{-6})	-	-
	Introduction of PPCI	0.63 (0.62 to 0.63)	-0.40 (-0.44 to -0.36)	1.69×10^{-4} (1.16×10^{-4} to 2.21×10^{-4})	-1.97×10^{-5} (-2.28×10^{-5} to -1.67×10^{-5})	-13.2 (-22.0 to -9.2)	<0.001
8	$P2Y_{12}$ inhibitors	0.79 (0.78 to 0.79)	-0.24 (-0.29 to -0.19)	-6.09×10^{-6} (-6.95×10^{-5} to 5.97×10^{-5})	-9.25×10^{-6} (-1.13×10^{-5} to -7.23×10^{-6})	-	-
	Introduction of PPCI	0.63 (0.62 to 0.63)	-0.40 (-0.44 to -0.36)	1.69×10^{-4} (1.16×10^{-4} to 2.21×10^{-4})	-1.97×10^{-5} (-2.28×10^{-5} to -1.67×10^{-5})	-13.2 (-22.0 to -9.2)	<0.001
9	$P2Y_{12}$ inhibitors	0.79 (0.78 to 0.79)	-0.24 (-0.29 to -0.19)	-2.85×10^{-6} (-6.64×10^{-5} to 6.28×10^{-5})	-9.31×10^{-6} (-1.13×10^{-5} to -7.27×10^{-6})	-	-

Dondo TB, *et al. Heart* 2020; 106:765–771. doi: 10.1136/heartjnl-2019-315760

Imputation dataset	Analysis	Intervention-mediator effect (path a_1 and a_2)	Mediator-outcome effect (path b_1 and b_2)	ADE	ACME	Proportion mediated (%)	P value
	Introduction of PPCI	0.63 (0.62 to 0.63)	-0.40 (-0.44 to -0.36)	1.70×10^{-4} (1.17×10^{-4} to 2.22×10^{-4})	-1.97×10^{-5} (-2.27×10^{-5} to -1.67×10^{-5})	-13.1 (-21.5 to -9.1)	<0.001
10	$P2Y_{12}$ inhibitors	0.79 (0.78 to 0.79)	-0.24 (-0.29 to -0.19)	-1.65×10^{-6} (-6.49×10^{-5} to 6.39×10^{-5})	-9.34×10^{-6} (-1.13×10^{-5} to -7.29×10^{-6})	-	-
	Introduction of PPCI	0.63 (0.62 to 0.63)	-0.40 (-0.44 to -0.36)	1.70×10^{-4} (1.17×10^{-4} to 2.22×10^{-4})	-1.95×10^{-5} (-2.26×10^{-5} to -1.66×10^{-5})	-13.0 (-21.3 to -9.2)	<0.001

Abbreviations: PPCI – primary percutaneous coronary intervention. *Complete case analysis results.

Dondo TB, *et al. Heart* 2020; 106:765–771. doi: 10.1136/heartjnl-2019-315760

eTable 7. Mediation analysis modelling for six months survival, by imputation dataset.

Imputation dataset	Analysis	Intervention-mediator effect (path a_1 and a_2)	Mediator-outcome effect (path b_1 and b_2)	ADE	ACME	Proportion mediated (%)	P value
0*	$P2Y_{12}$ inhibitors	-0.07 (-0.08 to -0.05)	-0.02 (-0.15 to 0.11)	$1.48{\times}10^{-4}$ ($-1.97{\times}10^{-5}$ to $2.66{\times}10^{-4}$)	$8.31{\times}10^{-7}$ ($-6.81{\times}10^{-6}$ to $8.83{\times}10^{-6}$)	-	-
	Introduction of PPCI	0.32 (0.31 to 0.34)	-0.39 (-0.47 to -0.32)	$1.69{\times}10^{-4}$ ($-4.29{\times}10^{-5}$ to $3.10{\times}10^{-4}$)	$-8.47{\times}10^{-5}$ ($-1.16{\times}10^{-4}$ to $-6.10{\times}10^{-5}$)	-	-
1	$P2Y_{12}$ inhibitors	0.79 (0.78 to 0.79)	-0.23 (-0.29 to -0.17)	$2.05{\times}10^{-4}$ ($1.17{\times}10^{-4}$ to $2.90{\times}10^{-4}$)	$-1.01{\times}10^{-5}$ ($-1.29{\times}10^{-5}$ to $-7.38{\times}10^{-6}$)	-5.2 (-8.7 to -3.5)	<0.001
	Introduction of PPCI	0.63 (0.62 to 0.63)	-0.43 (-0.48 to -0.39)	$2.16{\times}10^{-4}$ ($1.34{\times}10^{-4}$ to $2.99{\times}10^{-4}$)	$-3.07{\times}10^{-5}$ ($-3.60{\times}10^{-5}$ to $-2.56{\times}10^{-5}$)	-16.7 (-31.2 to -10.8)	<0.001
2	$P2Y_{12}$ inhibitors	0.79 (0.78 to 0.79)	-0.23 (-0.29 to -0.17)	$2.06{\times}10^{-4}$ ($1.17{\times}10^{-4}$ to $2.91{\times}10^{-4}$)	$-1.02{\times}10^{-5}$ ($-1.29{\times}10^{-5}$ to $-7.42{\times}10^{-6}$)	-5.2 (-8.6 to -3.4)	<0.001

Dondo TB, *et al. Heart* 2020; 106:765–771. doi: 10.1136/heartjnl-2019-315760

**Imputation dataset**	**Analysis**	**Intervention-mediator effect (path a_1 and a_2)**	**Mediator-outcome effect (path b_1 and b_2)**	**ADE**	**ACME**	**Proportion mediated (%)**	**P value**
	Introduction of PPCI	0.63 (0.62 to 0.63)	-0.43 (-0.48 to -0.39)	2.17×10^{-4} (1.34×10^{-4} to 3.00×10^{-4})	-3.06×10^{-5} (-3.60×10^{-5} to -2.54×10^{-5})	-16.6 (-30.8 to -10.8)	<0.001
3	$P2Y_{12}$ inhibitors	0.79 (0.78 to 0.79)	-0.23 (-0.29 to -0.17)	2.02×10^{-4} (1.14×10^{-4} to 2.88×10^{-4})	-1.01×10^{-5} (-1.29×10^{-5} to -7.35×10^{-6})	-5.3 (-8.9 to -3.4)	<0.001
	Introduction of PPCI	0.63 (0.62 to 0.63)	-0.43 (-0.48 to -0.38)	2.13×10^{-4} (1.30×10^{-4} to 2.96×10^{-4})	-3.06×10^{-5} (-3.58×10^{-5} to -2.55×10^{-5})	-17.0 (-31.8 to 11.0)	<0.001
4	$P2Y_{12}$ inhibitors	0.79 (0.78 to 0.79)	-0.23 (-0.29 to -0.17)	1.98×10^{-4} (1.09×10^{-4} to 2.83×10^{-4})	-1.01×10^{-5} (-1.29×10^{-5} to -7.31×10^{-6})	-5.3 (-9.2 to -3.6)	<0.001
	Introduction of PPCI	0.62 (0.62 to 0.63)	-0.43 (-0.48 to -0.39)	2.08×10^{-4} (1.25×10^{-4} to 2.92×10^{-4})	-3.08×10^{-5} (-3.60×10^{-5} to -2.56×10^{-5})	-17.4 (-34.3 to -11.2)	<0.001
5	$P2Y_{12}$ inhibitors	0.79 (0.78 to 0.79)	-0.23 (-0.29 to -0.17)	2.02×10^{-4} (1.13×10^{-4} to 2.87×10^{-4})	-1.01×10^{-5} (-1.30×10^{-5} to -7.40×10^{-6})	-5.3 (-8.9 to -3.6)	<0.001

Dondo TB, *et al. Heart* 2020; 106:765–771. doi: 10.1136/heartjnl-2019-315760

Imputation dataset	Analysis	Intervention-mediator effect (path a_1 and a_2)	Mediator-outcome effect (path b_1 and b_2)	ADE	ACME	Proportion mediated (%)	P value
	Introduction of PPCI	0.63 (0.62 to 0.63)	-0.43 (-0.48 to -0.38)	2.13×10^{-4} (1.30×10^{-4} to 2.96×10^{-4})	-3.06×10^{-5} (-3.60×10^{-5} to -2.53×10^{-5})	-17.0 (-31.9 to -11.0)	<0.001
6	$P2Y_{12}$ inhibitors	0.79 (0.78 to 0.79)	-0.23 (-0.28 to -0.17)	2.04×10^{-4} (1.16×10^{-4} to 2.90×10^{-4})	-1.02×10^{-5} (-1.30×10^{-5} to -7.36×10^{-6})	-5.2 (-8.7 to -3.5)	<0.001
	Introduction of PPCI	0.63 (0.62 to 0.63)	-0.43 (-0.48 to -0.38)	2.15×10^{-4} (1.33×10^{-4} to 2.98×10^{-4})	-3.05×10^{-5} (-3.57×10^{-5} to -2.54×10^{-5})	-16.8 (-31.6 to -10.8)	<0.001
7	$P2Y_{12}$ inhibitors	0.79 (0.78 to 0.79)	-0.23 (-0.29 to -0.17)	2.05×10^{-4} (1.16×10^{-4} to 2.90×10^{-4})	-1.02×10^{-5} (-1.31×10^{-5} to -7.47×10^{-6})	-5.3 (-8.7 to -3.6)	<0.001
	Introduction of PPCI	0.63 (0.62 to 0.63)	-0.43 (-0.48 to -0.38)	2.15×10^{-4} (1.33×10^{-4} to 2.98×10^{-4})	-3.06×10^{-5} (-3.59×10^{-5} to -2.54×10^{-5})	-16.7 (-31.5 to -10.8)	<0.001
8	$P2Y_{12}$ inhibitors	0.79 (0.78 to 0.79)	-0.23 (-0.29 to -0.17)	2.05×10^{-4} (1.16×10^{-4} to 2.90×10^{-4})	-1.02×10^{-5} (-1.31×10^{-5} to -7.47×10^{-6})	-5.3 (-8.7 to -3.6)	<0.001

Dondo TB, *et al. Heart* 2020; 106:765–771. doi: 10.1136/heartjnl-2019-315760

**Imputation dataset**	**Analysis**	**Intervention-mediator effect (path a_1 and a_2)**	**Mediator-outcome effect (path b_1 and b_2)**	**ADE**	**ACME**	**Proportion mediated (%)**	**P value**
	Introduction of PPCI	0.63 (0.62 to 0.63)	-0.43 (-0.48 to -0.38)	2.15×10^{-4} (1.33×10^{-4} to 2.98×10^{-4})	-3.06×10^{-5} (-3.59×10^{-5} to -2.54×10^{-5})	-16.7 (-31.5 to -10.8)	<0.001
9	$P2Y_{12}$ inhibitors	0.79 (0.78 to 0.79)	-0.23 (-0.29 to -0.17)	2.01×10^{-4} (1.12×10^{-4} to 2.87×10^{-4})	-1.01×10^{-5} (-1.29×10^{-5} to -7.35×10^{-6})	-5.3 (-8.9 to -3.6)	<0.001
	Introduction of PPCI	0.63 (0.62 to 0.63)	-0.43 (-0.48 to -0.38)	2.11×10^{-4} (1.29×10^{-4} to 2.95×10^{-4})	-3.07×10^{-5} (-3.60×10^{-5} to -2.54×10^{-5})	-17.2 (-32.7 to -11.0)	<0.001
10	$P2Y_{12}$ inhibitors	0.79 (0.78 to 0.79)	-0.23 (-0.29 to -0.18)	2.05×10^{-4} (1.16×10^{-4} to 2.90×10^{-4})	-1.03×10^{-5} (-1.31×10^{-5} to -7.53×10^{-6})	-5.8 (-8.8 to -3.6)	<0.001
	Introduction of PPCI	0.63 (0.62 to 0.63)	-0.43 (-0.48 to -0.38)	2.15×10^{-4} (1.33×10^{-4} to 2.98×10^{-4})	-3.03×10^{-5} (-3.57×10^{-5} to -2.51×10^{-5})	-16.7 (-30.7 to -10.8)	<0.001

Abbreviations: PPCI – primary percutaneous coronary intervention. *Complete case analysis results.

eTable 8. Impact of patient and treatment factors on temporal trends in six months and one year survival between 2004 and 2013, for unadjusted and adjusted flexible parametric survival models (including in-hospital deaths) (complete case analysis, n=137,111).

		**Six months**		**One year**	
**Model number**	**Variables included**	**Hazard ratio (95% CI)**	**P value**	**Hazard ratio (95% CI)**	**P value**
Model 1	Year	0.96 (0.95-0.97)	<0.001	0.96 (0.95-0.97)	<0.001
	Year +				
Model 2	Age, sex, IMD	0.96 (0.95-0.96)	<0.001	0.96 (0.95-0.96)	<0.001
Model 3	Age, sex, IMD, Comorbidities and risk factors	0.96 (0.95-0.96)	<0.001	0.96 (0.95-0.96)	<0.001
Model 4	Age, sex, IMD, Comorbidities and risk factors, PPCI	1.02 (1.01-1.03)	<0.001	1.02 (1.01-1.03)	<0.001

Abbreviations: ACEi – angiotensin-converting enzyme inhibitor and ARBs – Angiotensin receptor blocker; IMD – index of multiple deprivation; PPCI – primary percutaneous coronary intervention.

Dondo TB, *et al. Heart* 2020; 106:765–771. doi: 10.1136/heartjnl-2019-315760

eTable 9. Mediation analysis modelling (including in-hospital deaths), by survival time (complete case analysis, n=137,111).

Survival time	Analysis	Intervention-mediator effect (path a_1 and a_2)	Mediator-outcome effect (path b_1 and b_2)	ADE	ACME	Proportion mediated (%)	P value
One year	Introduction of PPCI	0.60 (0.60-0.61)	-0.66 (-0.71 to -0.62)	0.006 (0.005 to 0.007)	-0.0007 (-0.0008 to -0.0006)	-12.7 (-16.6 to -10.0)	<0.001
Six months	Introduction of PPCI	0.60 (0.60-0.61)	-0.71 (-0.76 to -0.67)	5.68×10^{-4} (3.61×10^{-4} to 7.27×10^{-4})	-1.07×10^{-4} (-1.26×10^{-4} to -9.07×10^{-5})	-22.6 (-44.6 to -16.1)	<0.001

Abbreviations: PPCI – primary percutaneous coronary intervention.

Dondo TB, *et al. Heart* 2020; 106:765–771. doi: 10.1136/heartjnl-2019-315760

References

1 Tingley D, Yamamoto T, Hirose K, et al. Mediation: R package for causal mediation analysis. 2014.

2 Imai K, Keele L, Tingley D, et al. Causal mediation analysis using R. *Advances in social science research using R*: Springer 2010:129-54.

3 Imai K, Keele L, Yamamoto T. Identification, inference and sensitivity analysis for causal mediation effects. *Statistical science* 2010:51-71.

4 Linden A, Karlson KB. Using mediation analysis to identify causal mechanisms in disease management interventions. *Health Services and Outcomes Research Methodology* 2013;**13**:86-108.

5 Kenny DA. Mediation. Department of Psychological Sciences, University of Connecticut: http://davidakenny.net/cm/mediate.htm [Accessed 12 December 2017] 2015.

6 Cattle B, Baxter P, Greenwood D, et al. Multiple imputation for completion of a national clinical audit dataset. *Stat Med* 2011;**30**:2736-53.

7 Somma KA, Bhatt DL, Fonarow GC, et al. Guideline adherence after ST-segment elevation versus non-ST segment elevation myocardial infarction. *Circ Cardiovasc Qual Outcomes* 2012;**5**:654-61.

Dondo TB, *et al. Heart* 2020; 106:765–771. doi: 10.1136/heartjnl-2019-315760
